# Does size matter? The influence of shoe-hole sizes on foot-mounted marker motion during walking gait

**DOI:** 10.1186/1757-1146-7-S1-A7

**Published:** 2014-04-08

**Authors:** Chris Bishop, John B Arnold, Francois Fraysse, Dominic Thewlis

**Affiliations:** 1 Biomechanics and Neuromotor Laboratory, School of Health Sciences, University of South Australia, Australia; 2 Sansom Institute for Health Research, University of South Australia, Australia; 3 Centre for Orthopaedic and Trauma Research, University of Adelaide, Australia

## Background

To quantify in-shoe foot kinematics, studies have relied on cutting holes in the shoe upper to allow markers to be placed on the foot. Although previous research has suggested optimum hole sizes to preserve the structural integrity of the shoe [1], there is no empirical basis for what size holes are required to allow free-motion of individual markers during gait. The aim of this study was to determine the effect of different diameter holes on skin-mounted marker motion during walking in athletic footwear.

## Methods

Eighteen healthy adults participated in this study (10M:8F, mean age 22.7 years SD 3.7, height 1.74 m SD 0.08, body mass 71.2 kg SD 8.5). Wand-mounted surface markers were attached directly to the foot [2] or directly to the foot for barefoot measurements, which were used as a reference for comparisons. Each participant performed five walking trials in athletic footwear (ASICS Gel-Pulse 3). Three sets of identical shoes were used with holes of 15 mm (A), 20 mm (B) and 25 mm (C). All conditions were tested in a random order. Marker trajectories were acquired with 12 VICON cameras (MX-F20, VICON, UK) at 100 Hz. Data analysis was conducted in two parts; firstly, the movement (marker trajectory) of individual markers relative to the origin of a fixed shoe reference frame was quantified. Secondly, we adapted a method proposed by Cappozzo et al. [3] quantified the isotropy of the marker motion on a plane.

## Results

Where movement of the markers in the 15 and 20 mm conditions were restricted by the surrounding shoe upper, the marker movement in the 25 mm condition did not exceed the radius of any of the shoe-holes. Despite significant differences in the isotropy index between 25 mm and barefoot at the medial and lateral calcaneus markers (*P* < 0.05), the differences identified were due to the effect of footwear on the foot and not a result of the marker wands hitting the shoe upper.

**Figure 1:**
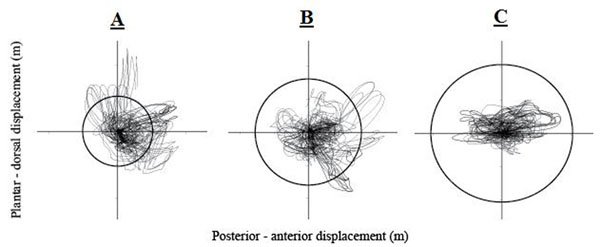
Example X-Y trajectory plots with ellipses to represent the radius of each shoe holes size for Calc 1 marker.

## Conclusion

When quantifying in-shoe foot kinematics, the size of the holes cut in the shoe upper can have a significant impact on the motion of surface markers attached to the foot. Using the methods in this study, it appears hole diameters smaller than 25 mm resulted in a restriction of surface marker motion, which may impact upon the resultant joint kinematics.
